# Biomining of MoS_2_ with Peptide-based Smart Biomaterials

**DOI:** 10.1038/s41598-018-21692-4

**Published:** 2018-02-20

**Authors:** Sibel Cetinel, Wei-Zheng Shen, Maral Aminpour, Prasanna Bhomkar, Feng Wang, Elham Rafie Borujeny, Kumakshi Sharma, Niloofar Nayebi, Carlo Montemagno

**Affiliations:** 1 Ingenuity Lab, 11421 Saskatchewan Drive, T6G 2M9 Edmonton, AB Canada; 2 Department of Chemical and Materials Engineering, University of Alberta, T6G 2V4 Edmonton, AB Canada; 3 National Institute of Nanotechnology (NINT), University of Alberta, T6G 2M9 Edmonton, AB Canada; 4 Southern Illinois University, 62901 Carbondale, IL USA

## Abstract

Biomining of valuable metals using a target specific approach promises increased purification yields and decreased cost. Target specificity can be implemented with proteins/peptides, the biological molecules, responsible from various structural and functional pathways in living organisms by virtue of their specific recognition abilities towards both organic and inorganic materials. Phage display libraries are used to identify peptide biomolecules capable of specifically recognizing and binding organic/inorganic materials of interest with high affinities. Using combinatorial approaches, these molecular recognition elements can be converted into smart hybrid biomaterials and harnessed for biotechnological applications. Herein, we used a commercially available phage-display library to identify peptides with specific binding affinity to molybdenite (MoS_2_) and used them to decorate magnetic NPs. These peptide-coupled NPs could capture MoS_2_ under a variety of environmental conditions. The same batch of NPs could be re-used multiple times to harvest MoS_2_, clearly suggesting that this hybrid material was robust and recyclable. The advantages of this smart hybrid biomaterial with respect to its MoS_2_-binding specificity, robust performance under environmentally challenging conditions and its recyclability suggests its potential application in harvesting MoS_2_ from tailing ponds and downstream mining processes.

## Introduction

Valuable and rare earth metals exist in trace amounts as a by-product of ore processing. However, the recovery of these rare earth minerals is currently not feasible because of extensive downstream purification processes resulting in the loss of valuable resource. These metals also accumulate in tailing ponds, causing extensive environmental pollution. Therefore, an environmentally sustainable and effective approach is necessary to facilitate their recovery from acid mine drainages, tailing ponds and polluted water sources.

Tremendous progress in several inter-disciplinary fields of biotechnology offers unprecedented opportunities to develop innovative solutions for several problems plaguing the mining industry. Under the term *biomining*, microorganisms are exploited for their ability to extract metal cations through biological oxidation using enzymatic processes^1–4^. Alternatively, microorganisms displaying specific protein sequences on their surfaces enable extraction by conducing metal chelation^5–7^. Similarly, bacteriophages displaying specific peptides on their coat allow selective recognition of metals and their recovery by aggregation and/or flocculation^8–10^.

Recently, we have generated an alternative biomining approach based on the utilization of peptide-functionalized magnetic nanoparticles^11^. Therein, the biomining of gold colloids was demonstrated without the need for excessive downstream solvent extraction, filtration, and concentration steps. The smart biomaterial designed for biomining applications was composed of a gold-binding peptide coated on a magnetic nanoparticle (NP) core. This hybrid system enabled the specific recognition and binding to gold through the peptide region and recovery of the metal from the solution with an external magnetic field. Additionally, this approach allowed us to work in mild conditions and obviate hazardous chemicals.

In this manuscript, we demonstrate the potential application of smart biomaterials for biomining of molybdenite (MoS_2_). Although there are mines that produce primarily molybdenite, significant amount of MoS_2_ is also found as a trace element in copper and tungsten mines. Besides its main application as a dry lubricant and as a catalyst for desulfurization, molybdenite can be used in the fabrication of ultrasensitive and switchable transistors, light emitting diodes, flexible solar cells and actuators^12,13^. Due to the broadening application areas, there is an increasing worldwide demand for molybdenum. Biomining may provide a convenient and efficient tool for molybdenite recovery and thus contribute to meeting the ever-increasing demand of this rare metal.

Herein, we selected MoS_2_ binding peptides from a phage display peptide library and analyzed two strong, one weak binders with molecular modeling studies to reveal the surface interacting residues. Two of those peptides are investigated further to characterize their binding affinity, specificity and recovery (elution) behaviors. Eventually, the best peptide candidate for biomining applications is used to decorate the magnetic nanoparticles. These smart biomaterials (magnetic nanoparticles coated with the MoS_2_-binding peptides) are shown to successfully capture and pull down MoS_2_ from aqueous solutions.

## Methods

### Phage Display and Peptide Selection

MoS_2_ binding peptides were selected from a Ph.D-12 phage display peptide library (New England Biolabs, Catalog # E8110S) by using 1 ml of 10^11^pfu/ml phage for the first round. MoS_2_ powder (~6 μm Sigma, Catalog # 69860) was cleaned by washing sequentially with acetone:methanol (1:1), isopropanol, and water, respectively and vacuum dried prior to use (Supplementary Figure S1). PBST buffer (150 mM NaCl, 2 mM KH_2_PO_4_, 16 mM Na_2_HPO_4_, 27 mM KCl pH 7.4) containing 0.1% Tween20/80 (1:1 ratio) was used for phage binding while detergent stringency was increased from 0.1% to a maximum of 0.5% for washing steps during subsequent biopanning rounds. Phage elution was achieved with EB1 (0.2 M glycine, 1 mg/mL BSA pH 2.2), EB2 (0.1 M glycine, 2 mg/mL BSA, 0.5 M NaCl, 50 mM DTT, 3.5 mM TCEP pH 2.2) and physical elution (sonication in PBST buffer). After three rounds of biopanning, selected phage clones were sequenced and 41 clones were further characterized by fluorescence microscopy and spectrophotometry (see Supplementary Methods and Figure S3).

### Peptide-MoS_2_ Binding Analysis

Two of the strong binding clones MoS2-P15 (GVIHRNDQWTAPGGG) and MoS2-P28 (DRWVARDPASIFGGG) were synthesized with solid phase peptide synthesis (by AAPPTEC, LLC.) with 93.8% and 96.51% purities, respectively (for peptide properties, see Supplementary Table S1). The weak binding peptide MoS2-P3 (SVMNTSTKDAIEGGG) was synthesized in our laboratories with 93% purity by using AAPPTEC Focus XC Solid Phase Peptide Synthesizer. The binding of strong binders were tested in 0.2, 0.4, and 0.8 mg/ml concentrations against 15, 30, and 45 mg MoS_2_ powder (see Supplementary Figure S4) while the weak binder was tested against 30 and 45 mg MoS_2_ powder.

For binding, peptides were incubated with the powder in PBS buffer on a rotary shaker for two hours at room temperature. Following the incubation period, the powder was centrifuged at 8000 rpm and the unbound peptide was recovered. Then, the settled powder was washed with 1 ml PBS while incubating in rotary shaker for 30 min. The unbound and washed peptide solutions were measured with spectrophotometer (Agilent 8453 UV-Vis) at 220 nm to calculate the peptide concentration. A standard curve with five concentrations (0.1, 0.2, 0.4, 0.8, 1 mg/ml) was used for each peptide to calculate the unbound and wash amount. The sum of unbound and wash concentrations was subtracted from the initial amount of the peptide to find the bound peptide concentration.

The 30 mg powder was found to be optimal for binding experiments against 0.2 mg/ml peptide and all further experiments were performed with the same powder-peptide ratio. MoS2-P15 affinity to MoS_2_ at various pH values was tested by performing the binding step in different buffers; citrate buffer was used to create the environment for pH 3.0–6.0, whereas PBS buffer was used for pH 5.8–8.0 range.

### Quart Crystal Microbalance Analysis

Q-sense E4 Quartz Crystal Microbalance with Dissipation monitoring (QCM-D, Biolin Scientific) was used with MoS_2_ coated sensor chips to investigate the peptide binding kinetics onto MoS_2_ surfaces. 1.5 ml of peptide solution in PBS buffer was pumped with 100 μl/min rate once the baseline was established. Following the sample injection, 1.5 ml PBS buffer was pumped to wash out the unbound peptides (desorption). For each peptide at least 4 different peptide concentrations (0.5–6.0 μM) were tested. The MoS_2_ surface was cleaned before reuse by washing with 1% SDS solution and treating under ozone for 20 minutes.

The data was then analyzed using the Langmuir adsorption model to calculate the binding constants *k*_*a*_, *k*_*d*_ and *K*_*eq*_ (see Supplementary Methods online for details).

### Cross-specificity Analysis

Peptide binding to MoS_2_, Mo, Graphite, MgO, SiO_2_, Al_2_O_3_, CaO, S, Fe_2_O_3_, Cu, Tungsten and Zn were evaluated by using approximately the same surface area (using 30 mg MoS_2_ powder) of each material (Supplementary Table S2) and 900 μl of 0.2 mg/ml peptide. The incubation of the peptide with the materials was completed in 2 hours on a rotary shaker at room temperature. After the removal of supernatant (representing the unbound peptide fraction), the material pellet was washed with 1 ml PBS followed by a 10-minute incubation period. The peptides present in the unbound and washed fractions were measured by spectrophotometer at 220 nm to calculate the bound peptide concentration.

### Peptide Elution

1 ml of 0.2 mg/ml peptide was incubated with 30 mg of clean MoS_2_ powder for 2 hours at room temperature on a rotator. At the end of the binding period, the unbound peptide fraction was removed and the powder was washed with 1 ml PBS. In order to elute the peptides, 1 ml EB1 (0.2 M glycine, 1 mg/mL BSA, pH 2.2) was added and incubated at room temperature, on a rotator for 2 hours. Elution fraction was measured at 220 nm for the presence of peptide, using EB1 as the blank.

Alternative elution systems of 4 M NaCl, sonication (1 min and 5 min) and temperature (37 °C, 50 °C, and 60 °C) in PBS buffer were tested following the same experimental procedure.

### Molecular Dynamic Simulations

#### Models and Setup

Models of P15, P28 and P3 peptides, water molecules (TIP3P model), MoS_2_ surface were prepared in all-atom resolution using VMD software. The C-terminus of peptides was capped with an acetyl group (COCH_3_) to meet the experimental conditions. For each peptide, different initial conformations including β-strand, α-helix, and random coils were prepared. Models of pre-equilibrated liquid water were obtained by molecular dynamics simulation in a 3D periodic box in the NPT ensemble. All components of the simulations were performed in neutral pH. The thickness of the surface is two layers with lattice parameter of a = 3.16 and c = 12.29. The x and y dimensions of the simulation boxes were 54 Å and 55 Å, respectively while the value of the z dimension was adjusted to maintain the thickness of the metal slab and a constant density of 1000 kg/m^3^ of the supernatant aqueous phase containing water and the peptide. All simulation boxes met the 3D periodic boundary condition. For the preparation of the surface-peptide-water systems, the peptides with different initial conformations and with 12 different initial orientations relative to the surface (prepared by consecutive 30° rotation around X- and Y-axis of the peptide) were positioned close to the surface (closest contact ~3 Å) to enhance conformation sampling. A typical system in these simulations contains total 18589 atoms, 159 peptide atoms, 2039 MoS_2_ atoms, 16390 water atoms and 1 ion.

#### Force Field

For MoS_2_ surface, a Mo–S interatomic potential that combines a many-body reactive empirical bond-order (REBO) potential^14^ for covalently bonded Mo and S in each layer with a two-body Lennard-Jones (LJ) potential between the layers was used. The parameters for MoS_2_ were adopted from Liang *et al*.^15^ and implemented using LAMMPS software by Stewart *et al*.^16^. CHARMM36 force field^17^ parameters were used for peptides. For the interaction between MoS_2_ and peptides, water and ions, arithmetic mixing rules were applied. Recently, the same force field parameters were used to study bio-MoS_2_ interface Heiranian, *et al*.^18,19^.

#### Simulation Protocol

MD simulations of peptide-MoS_2_ surface binding were performed using the LAMMPS simulation package^20^. The binding energies were calculated as E_(Ads)_ = E_1_−E_2_ + E_3_−E_4_, which is the average energy of four systems: surface−peptide−solvent system (E_1_), the peptide−solvent system (E_2_), the solvent system (E_3_), and the surface−solvent system (E_4_)^21^. All energies were calculated under NVT condition in the production stage after the system reached equilibration. The SHAKE algorithm^22^ was used to maintain the rigidity of the water molecules. The long-range electrostatic interactions were computed by using the particle-particle-particle-mesh (PPPM) method with a time step of 1 fs. The cutoff distance for the LJ interactions was 12 Å. The systems were then equilibrated for 1 ns with NPT ensemble at 1 atm pressure and 300 K° temperature. NPT simulation ensured that the water concentration was equal to the bulk value of 1000 kg/m^3^. NVT simulation was performed for each peptide configuration until the system was equilibrated. For each configuration 2 ns NVT simulation was used to record trajectory after equilibration. Total time to sample each peptide configuration on surface was about 70 ns. The temperature was maintained at 300 K° by applying the Nosè-Hoover thermostat with a time constant of 0.1 ps. During simulations, the total energy and force interaction between each residue and the surface were calculated.

### Synthesis of Magnetic Nanoclusters Coated with MoS2-P15

Highly uniform quasi-spherical iron oxide magnetic nanoclusters (MNCs) (~300 nm) were synthesized by using a hydrothermal reaction with a slight modification^23^. Briefly, 0.5 g of FeCl_3_ and 0.2 g of NaOH were dissolved in 30 mL of ethylene glycol (EG) at 50 °C to obtain an orange-yellow suspension. Then, the suspension was transferred into a 100 mL polyphenyl (PPL) chamber in a stainless steel autoclave reactor. The autoclave reactor was sealed and heated to 240 °C for 4.5 hours, with an additional 2.5 hours to cool down to room temperature. The MNCs formed as a black precipitate in the PPL chamber. The collected MNCs were magnetically separated from the reaction mixture and washed thoroughly with ethanol (five times) and finally freeze-dried, yielding 240 mg of MNCs powder.

Then, MNCs were amine functionalized (MNC@APTES) for peptide conjugation. Briefly, 30 mg Fe_3_O_4_ based magnetic nanoparticle clusters (MNCs, ~300 nm) were dispersed in 200 ml isopropyl alcohol (IPA) with 2 ml H_2_O and 1 ml NH_3_·H_2_O by using a sonication bath. Then 0.5 g 3-aminopropyl)triethoxysilane (APTES) was added drop by drop in a 30-min duration. The reaction continued for 3 hours under sonication at 50 °C. No nanoparticle aggregation was observed during this time. The resulting MNC@APTES were washed five times in ethanol and five times in water and then freeze-dried. The functionalization of NH_2_ was quantified by a colorimetric assay of amine density, utilizing 4-nitrobenzoaldehyde (4-NBA), to be 1.04 NH_2_/nm^2,24^.

MNC@APTES@MoS2-P15 was prepared by using The Fmoc-MoS2-P15 (Fluorenylmethyloxycarbonyl-NH_2_-GVIHRNDQWTAPGGG) peptide with 91.9% purity (obtained from AAPPTEC, LLC.). 9.98 mg MNC@APTES was dispersed in anhydrous 1 ml DMF (*N*, *N*-dimethylformamide) with 10 minute sonication. After 5.62 mg Fmoc-MoS2-P15, 6 mg HCTU ((2-(6-Chloro-1H-benzotriazole-1-yl)−1,1,3,3-tetramethylaminium hexafluorophosphate), and 8 μl DIEA (*N*,*N*-Diisopropylethylamine) were added, the reaction was left on a shaker for 2 days. The resulting NPs were washed with 5 × 1 ml DMF and 5 × 1 ml DCM (dichloromethane). Then 1 ml 5% acetic anhydride and 1% DIEA in DMF were added to both MNC@APTES (a control) and MNC@APTES@Fmoc-MoS2-P15, shaking for 30 minutes at room temperature, to block the unreacted -NH2. The resulting nanoparticle colloids were washed 5 × 1 ml DMF and 5 × 1 ml DCM. A Kaiser test showed yellow, indicating that the NH_2_ groups were completely blocked in the reaction^25^. To de-protect the peptide, 1 ml of 20% piperidine in DMF was applied to the NPs for twice, each for 10 minutes. Washing was performed by 5 × 1 ml DMF and another 5 × 1 ml DCM and freeze-dried. The Kaiser test was performed again resulting in MNC@APTES showing yellow, while MNC@APTES@MoS2-P15 showing blue, indicating a successful peptide conjugation reaction.

A detailed characterization of MNCs was completed by applying SEM, TEM, X-ray powder diffraction (XRD), X-ray photoelectron spectra (XPS), Fourier Transform IR (FTIR), Magnetometer and finally BCA for quantification of peptide functionalization (see Supplementary Methods for details).

### MoS_2_ Binding and Elution with Magnetic Nanoclusters Coated with MoS2-P15

0.2 mg of MoS_2_ powder was dispersed in 500 μl Milli-Q water (pH 6.9). 1 mg of MNCs (in 500 μl Milli-Q water) with different surface functionalization was added into MoS_2_ solution. The mixture was incubated on shaker for 15 minutes before the MNCs were settled at the bottom using a magnet. After discarding the supernatant, the pull-down solids were digested in 20 mL Aqua Regia overnight in order to be completely dissolved and subsequently sent for ICP-MS measurement. To test the elution efficiency, the pulled down solid (MNCs bound to MoS_2_) was incubated in 1 ml EB1 on a shaker for 2 hours in order to disrupt the interaction between MNCs and MoS_2_. Following the incubation, supernatant (containing MoS_2_ if the elution was successful) was removed and pulled down MNCs were digested in 20 mL Aqua Regia before submitted for ICP-MS measurement.

The binding analysis was repeated with 1 mg of Al_2_O_3_ powder in order to observe the cross-specificity of MNCs.

## Results and Discussion

### Selection and Characterization of MoS_2_ Binding Phage Clones

12-mer peptides were selected against MoS_2_ powder following a 3-hour room temperature incubation of Ph.D-12 phage display peptide library with the material. Here, ~6 μm diameter size particles have been used in order to mimic the average 67 μm diameter size distribution of molybdenite concentrate^26,27^. Three panning rounds were performed to select peptide sequences with high binding affinity to MoS_2_. Analysis of selected sequences indicated the enrichment of non-polar amino acids as well as serine, tyrosine, histidine and aspartic acid (see Supplementary Figure S2). Initial semi-quantitative binding analysis of the phage clones was performed with immunolabeling. Briefly, phage clones were labeled with anti-M13/Alexa-Fluor antibodies and their relative surface coverage was measured as an indication of surface binding, directly from fluorescent microscopy images (examples are given in Supplementary Figure S3). Calculated relative binding affinities were also confirmed with label-free measurement of bound phage concentration/mg MoS_2_ powder using spectroscopy measurements. Here, the unbound phage concentration was calculated with the equation based on the relation between surface proteins and DNA content of the phage particles and utilized to calculate bound phage concentration^28^. The relative binding affinities of individual clones were found between 35–95% according to spectroscopy measurements and the clones exhibiting higher than 80% binding were considered to be strong binders. The wild type phage (without any insert peptide) was used as negative control and showed ~18% binding towards MoS_2_ powder.

Among the 41 selected phage clones, 10 were categorized as strong binders (>80% binding). Two of those strong binders, MoS2-P15 (GVIHRNDQWTAPGGG) and MoS2-P28 (DRWVARDPASIFGGG) as well as a weak binder MoS2-P3 (SVMNTSTKDAIEGGG) were synthesized with solid phase peptide synthesis and their MoS_2_ binding was further investigated.

### Binding characterizations of MoS2-P15 and MoS2-P28 peptides

**Figure 1:**
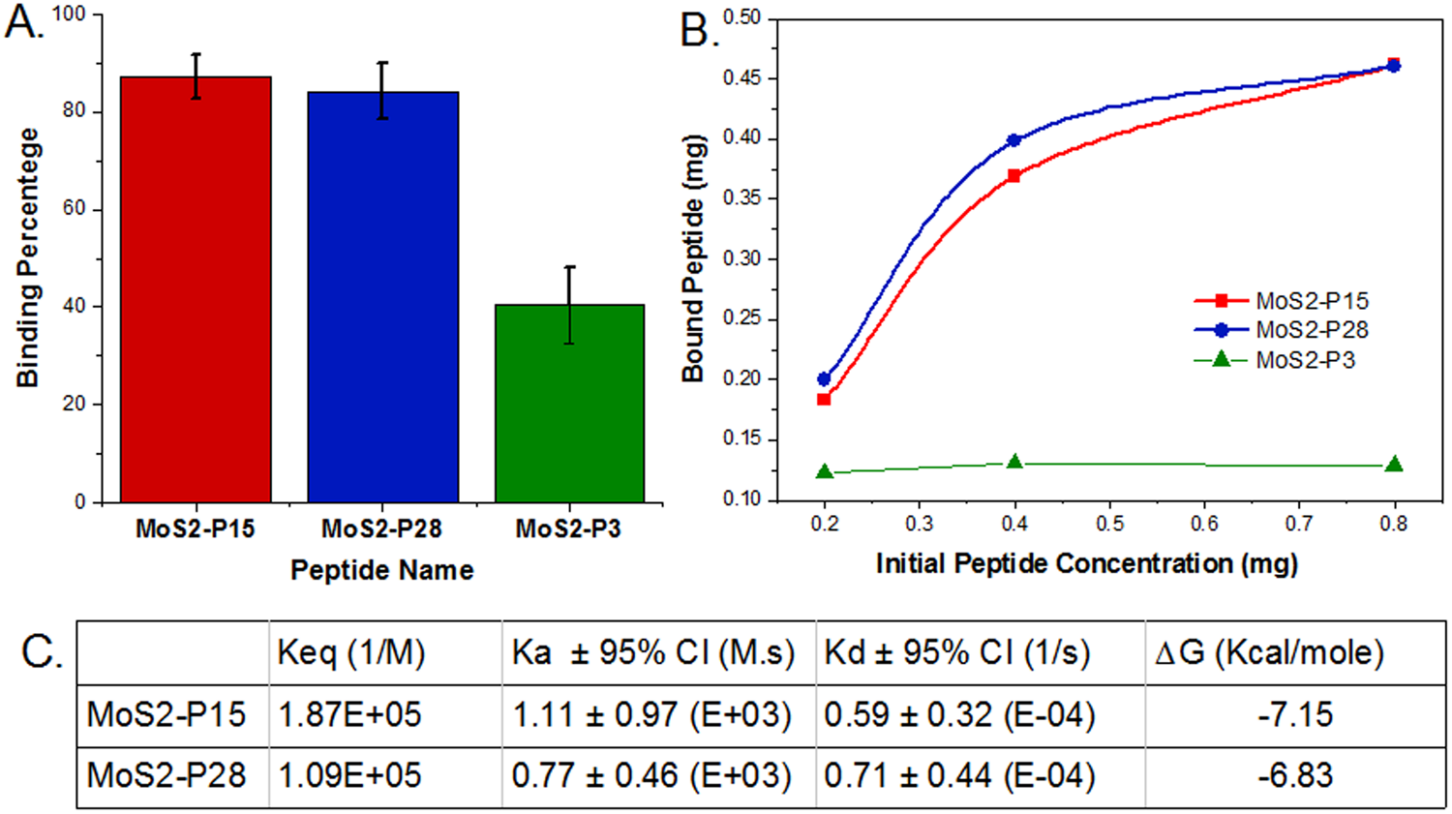
Binding analysis of MoS2-P15, MoS2-P28 and MoS2-P3 to MoS_2_ powder. (**A**) Peptide binding percentage of 0.2 mg peptide against 30 mg MoS_2_ powder. Data represent mean  ± S.D, n ≥ 3 (**B**) Binding trends of MoS2-P15, MoS2-P28 and MoS2-P3 with respect to increased peptide concentrations against 45 mg MoS_2_ powder (**C**) Binding kinetics of peptides derived from QCM analysis.

Each strong binding peptide was tested in 0.2, 0.4 and 0.8 mg/ml concentration against 15, 30, and 45 mg MoS_2_ powder. Almost no difference was observed when 15 mg powder was utilized, suggesting that the entire powder surface was covered with peptide (saturated) even at the lowest peptide concentrations (0.2 mg/ml). The surface area of the powder for 30 mg is ~5.9 × 10^15^ nm^2^. Assuming that one peptide molecule occupies 1 nm^2^ area, in case of full surface coverage, we estimate that there should be at least 5.9 × 10^15^ peptide molecules. Considering that all peptides are around 1.5 kDa, 0.2 mg peptide solution was estimated to contain 8 × 10^16^ peptide molecules, sufficient to cover the surface of 30 mg MoS_2_ powder. Any higher concentration or lower amount of powder would result in excess surface coverage, which might result in a multi-layer adsorption. Based on these considerations, 30 mg powder was found to be optimal for binding experiments with 0.2 mg/mL peptide, and all further experiments performed with the same powder-peptide ratio. The peptide binding strength was calculated as the bound peptide percentage (Fig. 1.A). While both strong binders showed ~80% binding affinity, the weak binder’s strength was only ~40%. Under these conditions, no significant difference among strong binders (MoS2-P15 and MoS2-P28) was measured.

However, when excess amount of powder (45 mg) was exposed to different peptide concentrations, it was possible to observe the peptides’ binding trends (Fig. 1.B). Nevertheless, the non-linear regression analysis for comparison of fits (with one way association) did not suggest different curve fittings for strong binders (p = 0.0524). Herein, K (rate constant) values for P15 and P28 were found to be 5.286 and 6.535 respectively (GraphPad, Prism 7.0c). Similarly, 2-way ANOVA analysis did not indicate a difference between binding trends of P15 versus P28, especially for the second half of the binding curve. These findings indicate a similar binding pattern for both MoS_2_ strong binding peptides.

Detailed quantitative kinetic constants of peptide binding were derived from QCM analysis for strong binders. Both peptides exhibited a self-driven binding pattern with negative free energy values, whereas MoS2-P15 (−7.15 kcal/mole) yielded a slightly lower binding energy compared to MoS2-P28 (−6.83 kcal/mole). Indicating a more favorable binding, the equilibrium constant of MoS2-P15 was also ~80% higher compared to MoS2-P28 (1.87 × 10^5^ and 1.09 × 10^5^ M^−1^, respectively). This difference is the result of relatively higher adsorption and lower desorption of MoS2-P15 (Fig. 1C). Finally, MoS2-P15 found to cover ~89% of the surface at 6 *μ*M, while MoS2-P28 reaches to only ~76% coverage at the same peptide concentration.

**Figure 2:**
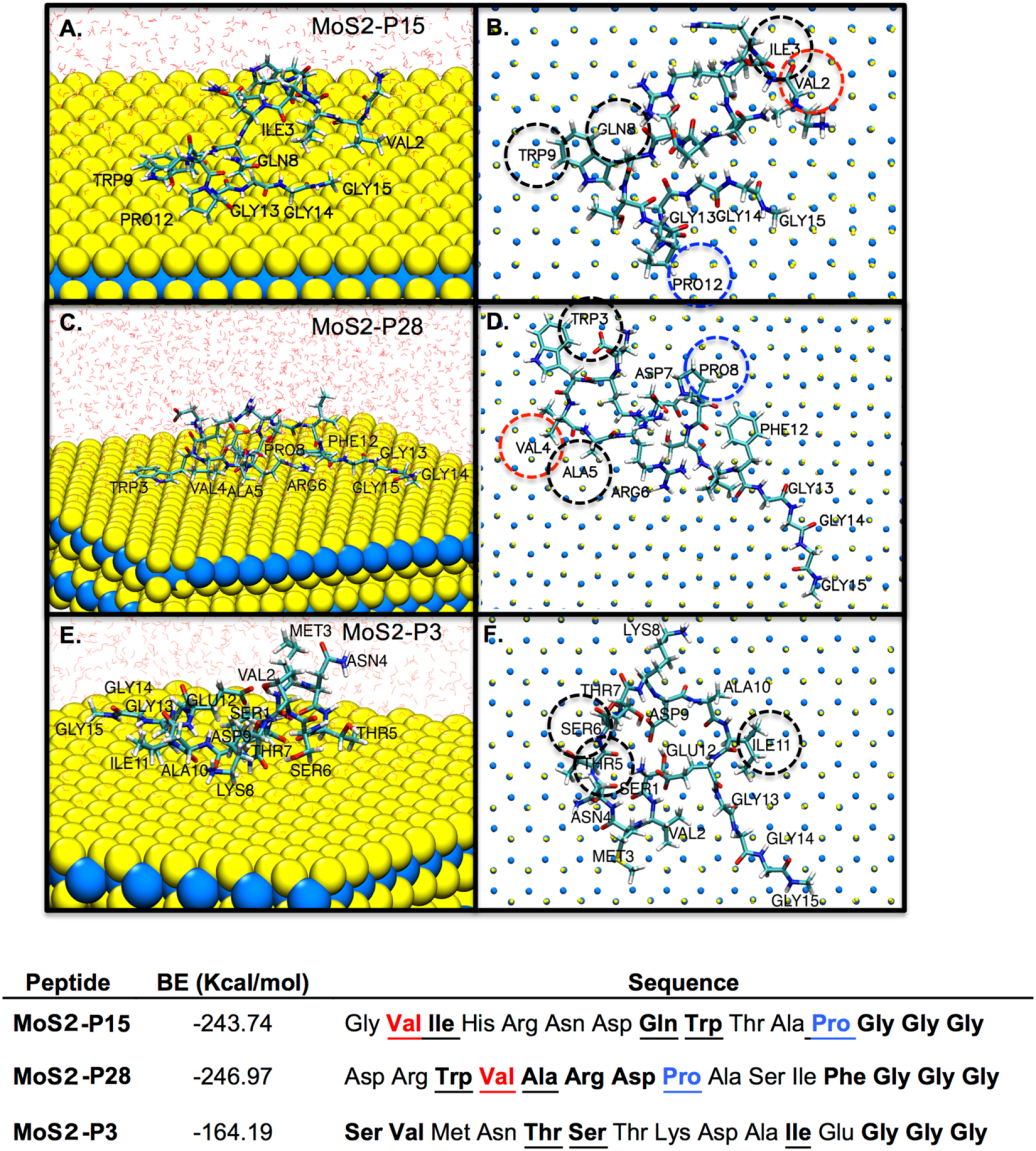
Molecular modeling trajectories of equilibrium conformations and adsorption energies of the peptides on MoS_2_ surface. (**A**–**F**) Top view and side view of MoS2-P15, MoS2-P28 and MoS2-P3 binding peptides on MoS_2_ surface. Surface binding residues were labeled with residue names and numbers. Dotted circles indicate configurations of the amino acids yielding to the strongest specific peptide interactions with MoS_2_ surface. Water molecules on side view were depicted as line representation, while omitted from the on top view for visual clarity. Peptide−MoS_2_ Adsorption Energies (kcal/mol) of MoS2-P15, MoS2-P28 and MoS2-P3 are given in the table.

The peptide adsorption to MoS_2_ surface was further studied using Molecular Dynamics (MD) simulations in order to identify the primary residues/regions responsible for surface recognition. For this purpose, all trajectories and equilibrium representative conformations of the peptides on the MoS_2_ surface were analyzed. Distances of specific residues from the surface in their equilibrium conformations were computed from coordinates in the recorded snapshots as an average over time. Consequently, peptide fragments and individual residues that contributed significantly to the adsorption energy were identified. The amino acids were considered as a strong interacting residue if their atoms exhibit more than 70% of the production run at a distance less than 3 Å (direct contact criteria) from the target surface (residues in bold in Fig. 2)^29^. The binding of those residues was either through their side-chain or backbone. To this end, the specific binders were considered as the residues that interact with the surface through their side chains in more than 70% of the production run (underlined residues in the table of Fig. 2 and circled residues in Fig. 2B,D and F).

Within calculation parameters, the specific surface interacting residues for MoS2-P15 were found to be Val (2), Ile (3), Gln (8), Trp (9) and Pro (12) (Fig. 2A,B). Similarly, Trp (3), Val (4), Ala (5) and Pro (8) residues were found to interact with the surface in MoS2-P28 (Fig. 2C,D). On the contrary, weak binder MoS2-P3 interacted with the surface through Thr (5), Ser (6) and Ile (11) (Fig. 2E,F) and exhibited a lower binding energy of −164.19 Kcal/mol compared to strong binders (−243.74 and −246.97 Kcal/mol for MoS2-P15 and MoS2-P28, respectively). Additionally, behaviour of MoS2-P3 trajectory on the surface is found to be distinct from that of the P15 and P28. Unlike the trajectories of the strong binders, which persist in extended conformations, MoS2-P3 prefers to be away from the surface and adopts more compact conformation.

In both strong binding peptides, Proline and Valine with strong hydrophobic properties were common as strong and specific interacting residues. The analysis of selected MoS_2_ binders has already revealed the relatively high abundance of Valine compared to initial library whereas Proline has been found to show relatively low abundance (see Supplementary Figure S2). The reason for this result might be arisen from the high prevalence of Proline in the initial library. Proline, due to its side chain wrap-around to form a covalent bond with the backbone, severely restricts the backbone conformation of neighboring residues^30^. Therefore, the presence of Proline in a peptide confers a higher structural constraint to the peptide that could facilitate Proline adhesion onto the surface^31^. The other common residue found in both peptides as surface interacting residue; Tryptophan exhibits an aromatic structure with an indole ring that contributes surface adhesion through π-π stacking interactions. The same phenomenon was already shown by GrBP5-M6 peptide (IMVTASSAYDDY), where “YDDY” region putatively binds to MoS_2_ through π-electrons of phenyl in Tyrosine and enhanced electronic interactions by two negatively charged Aspartates^32^. This peptide was also found to exhibit morphology of six-fold symmetry on the surface indicating the effect of hexagonal 2D lattice to peptide conformation.

Gaining insight about surface interacting residues for a given peptide group is very beneficial for further tailoring of the peptides. Therefore, the MD simulations are very valuable but limited to identify the binding confirmations of free peptides. Once the peptide is conjugated to the nanoparticle surface, a constraint will be introduced to the confirmation through the anchoring region. Additionally, nanoparticle surface in close proximity to the peptide may lead to the formation of lower energy configurations interacting with (resting on) the surface. Even though this affect might be eliminated with a careful control of peptide density on the surface, there is still a possibility that the configurations of peptide in free and anchored form might differ, which may result in altered binding affinities.

### Binding specificity and recovery of MoS2-P15 and MoS2-P28 peptides

**Figure 3:**
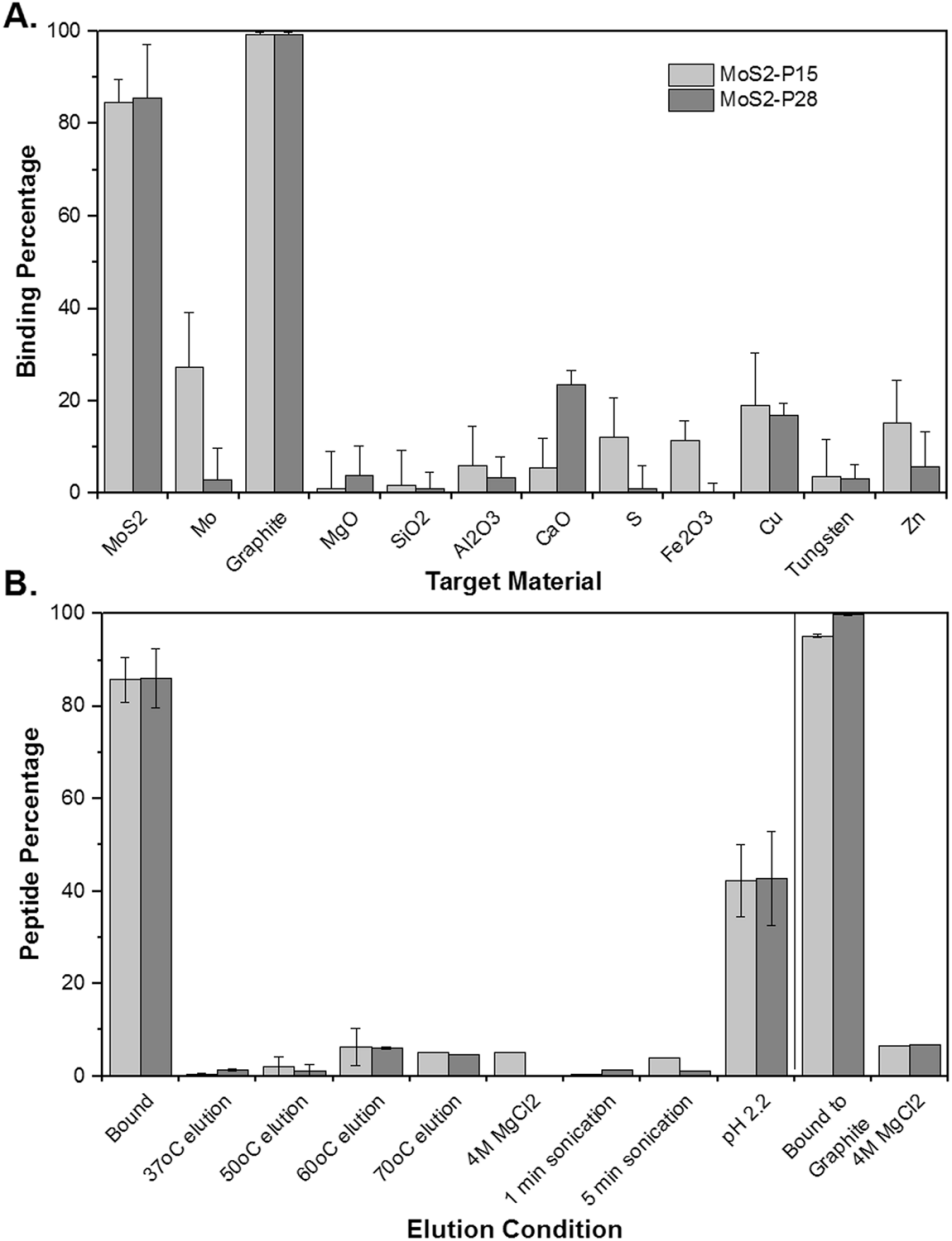
Cross-specificity and elution profiles of MoS2-P15 and MoS2-P28. (**A**) Peptide binding to MoS_2_, Mo, Graphite, MgO, SiO_2_, Al_2_O_3_, CaO, S, Fe_2_O_3_, Cu, Tungsten, Zn (Data represent mean ± S.D, n ≥ 3). (**B**) Elution from MoS_2_ and Graphite with different methods (Data represent mean ± S.D, n ≥ 2).

The removal of Mo and MoS_2_ from copper ores, copper ore slags, and tailing ponds is an important step in terms of protecting the environment through water treatment from tailing ponds, as well as recovering this valuable metal for various applications. In order to suggest peptides as recognition and capture elements for MoS_2_ mining from tailing ponds, their specificity becomes an important parameter. Therefore, the main components of copper slags – MoS_2_, Mo, MgO, SiO_2_, Al_2_O_3_, CaO, S, Fe_2_O_3_, Cu, Tungsten and Zn^33,34^ – were tested as possible targets for MoS2-P15 and MoS2-P28 (Fig. 3A). None of the peptides was found to exhibit a significant binding affinity to any of these materials, ultimately supporting the utilization of MoS2-P15 and MoS2-P28 as specific identifiers for MoS_2_ mining. Meanwhile, despite being a less abundant form in nature, the recognition of Mo by these peptides was also evaluated. Surprisingly, MoS2-P15 was able to recognize Mo with about 30% affinity, while MoS2-P28 bound to Mo with 5% affinity, indicating that MoS2-P15 is the better candidate for this particular biomining application.

Additionally, graphite was examined as a target due to its similar layered structure and two-dimensional form providing better electrical and optical properties than those found in three-dimensional forms of the materials. Both peptides exhibited almost 100% binding affinity to graphite, which was even greater than their affinity towards MoS_2_ (Fig. 3A). The driving force for graphite affinity may arise from the presence of hydrophobic residues as well as Tryptophan, which is known to be a critical residue in peptides that recognize graphene and graphite surfaces^35,36^. This result also supported the previous findings of GrBP5 peptide (IMVTESSDYSSY^37^) surface assembly on graphite and MoS_2_^32^. With the conserved aromatic residues of GrBP5, peptide was able to cover graphite surface with ~85% while covering MoS_2_ surface with ~80%. The film formed by the peptide on a single-layer of MoS_2_ was disorganized but confluent. The organized structures on MoS_2_ were maintained by creating a rational designed version, GrBP5-M6, by introducing negatively charged residues to bring the peptide closer to the surface. In this case, the peptide was able to cover MoS_2_ surface with 100% and graphite surface with 80%^32^. Similarly, the introduction of charged residues to MoS2-P15 and MoS2-P28 might be used to increase their affinity towards MoS_2_ in comparison to graphite.

The other parameter that is as important as specificity of smart biomaterial is its recovery, in other words recyclability. To use our technology in biomining application, it was essential to test whether the MoS_2_ can be eluted from the bound peptide, so that the metal can be processed (for downstream applications) and the peptide can be reused/recycled). For this purpose, recovery of the peptides from MoS_2_ powder was evaluated under different elution conditions. Any stringent conditions eliminating the molecular interactions are applicable for the elution process, such as the utilization of acidic buffers with a pH as low as 2.2, alkaline buffers such as 0.1 M triethylamine, denaturing buffers such as high concentration of urea (6 M, pH 3.0) and ionic buffers such as 4 M NaCl_2_^38–40^. Here a pH 2.2 buffer (which was already applied for elution processes during the panning rounds), 4 M MgCl_2_ buffer, temperature and physical elution were examined. Among the tested conditions, only the acidic condition resulted in the elution of the bound peptide (ca. 50% recovery) from MoS_2_ powder (Fig. 3B). Even though 50% is a limited yield for biomining applications, repetitive elution is likely to improve the efficiency of this process. On the other hand, peptide recovery from graphite under the same conditions was unsuccessful. Only 4 M MgCl_2_ resulted with <10% peptide recovery from graphite (Fig. 3B). In conclusion, these findings demonstrate that acidic conditions can be utilized for the recovery of peptide/MoS_2_ from the biomining process. Additionally, the presence of graphite in the processed slag wouldn’t interfere with the purity of recovered MoS_2_ since graphite won’t be eluted at acidic conditions applied for MoS_2_ elution.

### Effect of pH on MoS2-P15 Binding

**Figure 4:**
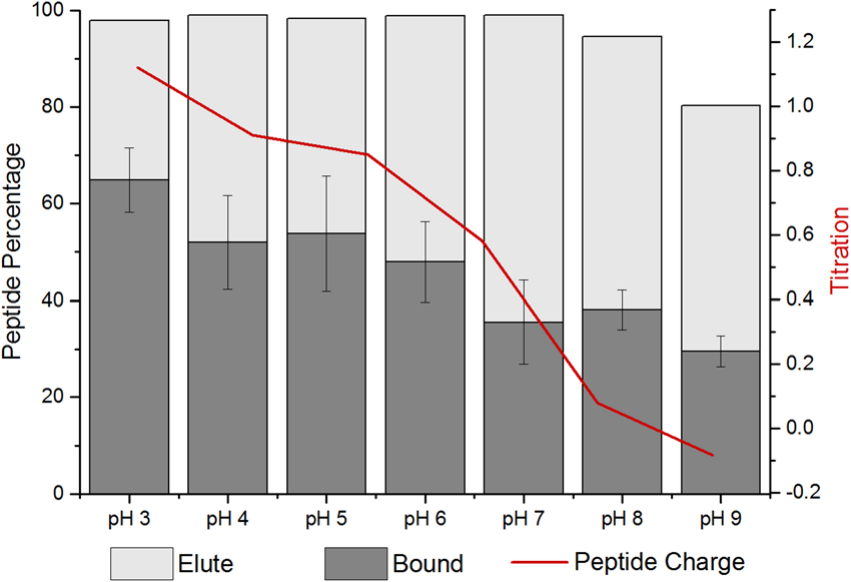
Binding trend of MoS2-P15 peptide in different environments with various pH and their elution efficiencies with pH 2.2 elution buffer (Data represent mean ± S.D, n ≥ 2).

Every acid mine drainage or tailing pond exhibit different chemical and physical characteristics based on the main ore and the mining processes. However inorganic binding peptides are being selected under certain buffer conditions and the interfacial forces between the peptide and the surface are susceptible to environmental factors. In order to provide a working window for MoS_2_ binding peptides in different environmental conditions, the binding regime and the stability (elution profile) of MoS2-P15 were studied under the absence of electrolytes and various pH conditions. Initially, peptide binding was evaluated in buffered solutions (PBS) as well as water. Similar binding affinities were observed regardless of whether the binding experiments were performed in a buffered environment or in water, indicating that there was no significant impact on the binding affinity in the presence or absence of electrolytes. Then, the peptide was incubated with the MoS_2_ powder in diverse buffer solutions with a pH range of 3.0–9.0. The bound peptide amount was measured initially and plotted with respect to the experimental pH (Fig. 4 – Dark + Light grey bars). Subsequently, the peptide was eluted using a highly acidic buffer (pH 2.2) and the recovered concentration from the surface was plotted against the experimental binding conditions (Fig. 4 – Light grey bar). The results indicated that MoS2-P15 binding to MoS_2_ was more favorable in neutral and acidic conditions. Shifting towards a basic state, binding affinity was found to have decreased (~20%). Contrarily, the peptide bound to MoS_2_ in basic conditions resulted in higher recovery (elution) percentage than the peptide bound to MoS_2_ in acidic conditions. Whereas high affinity and low recovery were postulated as surface stability of the peptide and drawn as a correlation with pI (isoelectric point) of the peptide, it was found that MoS2-P15 exhibited highest stability in protonated conformations. This finding, consistent with previous data, may suggest that charged residues play an active role in supporting MoS2-P15 surface interaction^32,36,37^. Conversely, the advanced stability of MoS2-P15 makes it a proper candidate for biomining applications in the harsh environments of tailing ponds and downstream mining processes.

### Synthesis of Magnetic Nanoclusters Coated with MoS2-P15

**Figure 5:**
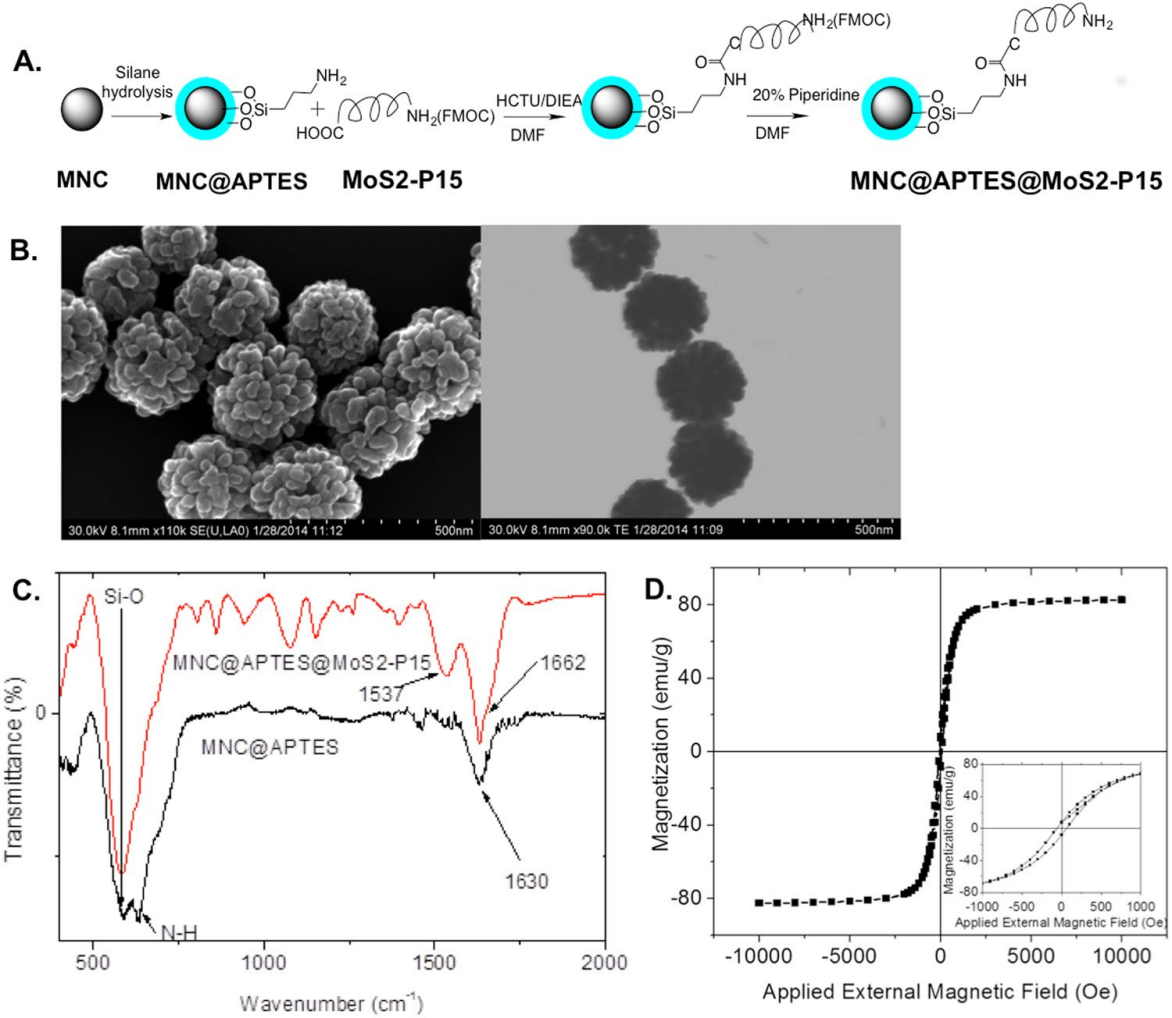
Synthesis and characterization of Magnetic Nanoclusters Coated with MoS2-P15. (**A**) Schematic illustration of the MNC@APTES@MoS2-P15 NP synthesis, (**B**) SEM and TEM images of MNC@APTES@MoS2-P15, (**C**) FTIR spectra of MNC@APTES and MNC@APTES@MoS2-P15, (**D**) Magnetic property of MNC@APTES@MoS2-P15.

Following the selective recognition of metal by the peptides, a carrier system should facilitate the separation of metal from the slurry such as induced flocculation or aggregation by the help of bacteriophage particles displaying the peptides^8,10^. Alternatively, peptides can be decorated on the surface of a magnetic nanoparticle and could be separated by using external magnetic field^11^. In this study, we applied the latter approach and prepared MoS2-P15 functionalized magnetic nanoclusters (Fig. 5A). In this three step protocol, first, highly uniformed spherical Fe_3_O_4_ based magnetic nanoclusters (MNCs) (~300 nm) were synthesized by using hydrothermal reaction with slight modification. Second, amine groups were introduced onto the surface of magnetic nanoclusters via silane hydrolysis and condensation. Third, the MoS2-P15 peptide with Fmoc protected N-terminal was introduced to the functionalized silane via coupling reaction. After the reaction, the remaining free amine groups from APTES were capped by acetic anhydride and Fmoc groups from MoS2-P15 were removed. Resulting MNC@APTES@MoS2-P15 yielded uniform structures according to SEM and TEM imaging (Fig. 5B). A Kaiser test was used to verify peptide conjugation reaction in each step, starting with a clear blue color indicating the presence of NH_2_ groups on the MNC@APTES surface. Following the coupling reaction of Fmoc-MoS2-P15 to MNC through the amide bond formation between the -COOH of Fmoc-MoS2-P15 and -NH_2_ of APTES with the sense that the unreacted NH_2_ groups of APTES was capped afterwards, the Kaiser test confirmed the reaction by showing light yellow. Eventually, Fmoc deprotection was confirmed by a blue Kaiser test result indicating the presence of deprotected MoS2-P15 peptide on the MCS surface.

Subsequently, grafting of MNCs with the peptide was evaluated using various characterization methods including FTIR and XPS. The FTIR spectra of MNC@APTES showed one peak at 550 cm^−1^ corresponding to the Si-O stretching. A second band at 600 cm^−1^ representing the N-H vibrating and a third peak at 1630 cm^−1^ corresponding to OH bending of the absorbed water was observed^41^. The spectrum of MNC@APTES@MoS2-P15 indicated two extra peaks compared to the spectrum of MNC@APTES, a sharp peak at 1662 cm^−1^ and a small shoulder at 1537 cm^−1^ corresponding to the C=O and C-N of the amide (HN-C=O) bond confirming the peptide conjugation on the nanoparticle surface (Fig. 5C).

On the other hand, high-resolution scans of XPS spectra (see Supplementary Figure S5) of C1 levels for both MNC@APTES and MNC@APTES@MoS2-P15 resulted with four peaks (C-C 285 eV, C-N 286.1 eV, O=C-NH 287.2 eV, O=C-OH 289.3 eV), in accordance with the literature. The C-C bond with binding energy of 285 eV exhibited a much higher intensity in MNC@APTES@MoS2-P15 than in MNC@APTES due to peptide conjugation. Additionally, the increased relative intensities of C-C, C-N/O and O=C-NH peaks clearly indicated the success of peptide conjugation on the nanoparticle surface.

Once peptide conjugation was confirmed, the magnetic properties of MNC@APTES@MoS2-P15 were evaluated by applying an external magnetic field and recording its responding magnetizations at room temperature. The MNC@APTES@MoS2-P15 showed a saturated magnetization of 86 emu/g, only slightly lower than the bulk iron oxide material (92 emu/g) (Fig. 5D).

Finally, the functionalization of NH_2_ was quantified to be 1/nm^2^ surface area by colorimetric assay of amine density utilizing 4-nitrobenzoaldehyde^41^. The conjugated MoS2-P15 peptides on MNC were quantified by BCA assay to be 40.5 *μ*g/mg (peptide/MNC).

The characterization studies on MNC@APTES@MoS2-P15 indicated that the MNC surface was perfectly coated with MoS2-P15 peptide and the result biomaterial exhibit ferromagnetic properties to facilitate their migration towards external magnetic field.

### MoS_2_ Recognition and Recovery with Magnetic Nanoclusters Coated with MoS2-P15

**Figure 6:**
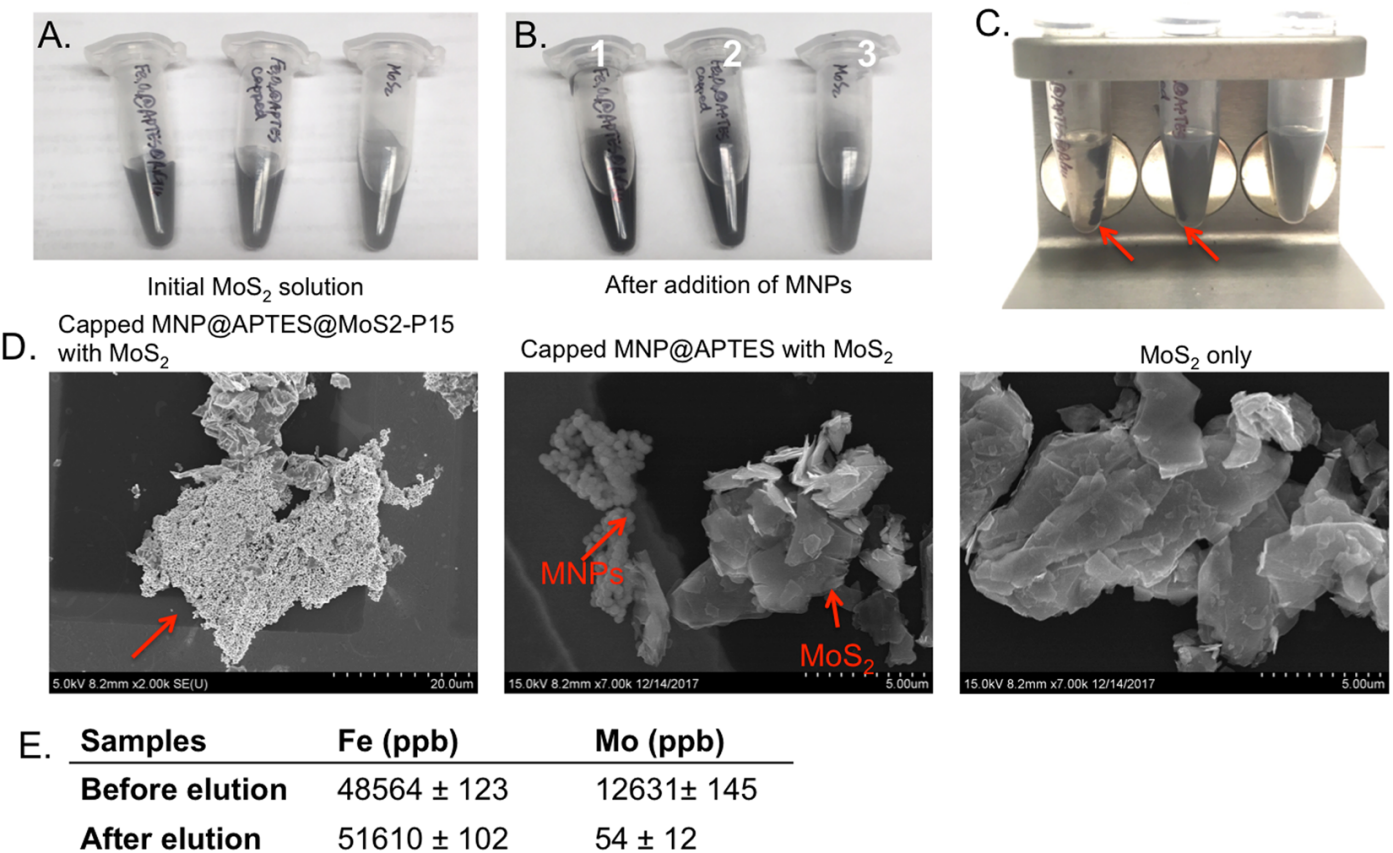
MoS_2_ recognition and recovery with Magnetic Nanoclusters coated with MoS2-P15. Picture of MoS_2_ binding test; (**A**) The tubes containing MoS_2_ powder, (**B**) The tubes containing MNC@APTES@MoS2-P15 with MoS_2_ powder (Tube-1), MNC@APTES with MoS_2_ powder (Tube-2) and MoS_2_ powder alone (Tube-3), respectively from left to right, (**C**) with an applied magnet behind. The arrows indicate the MNC location due to magnetic pull-down to the wall of the tube. (**D**) SEM images of MNC@APTES@MoS2-P15 bound MoS_2_, MNC@APTES bound MoS_2_ and MoS_2_ powder only. (**E**) ICP-MS results of magnet pull-down solids of Fe_3_O_4_@APTES@MoS2-P15 and MoS_2_ interaction before and after elution.

**Figure 7:**
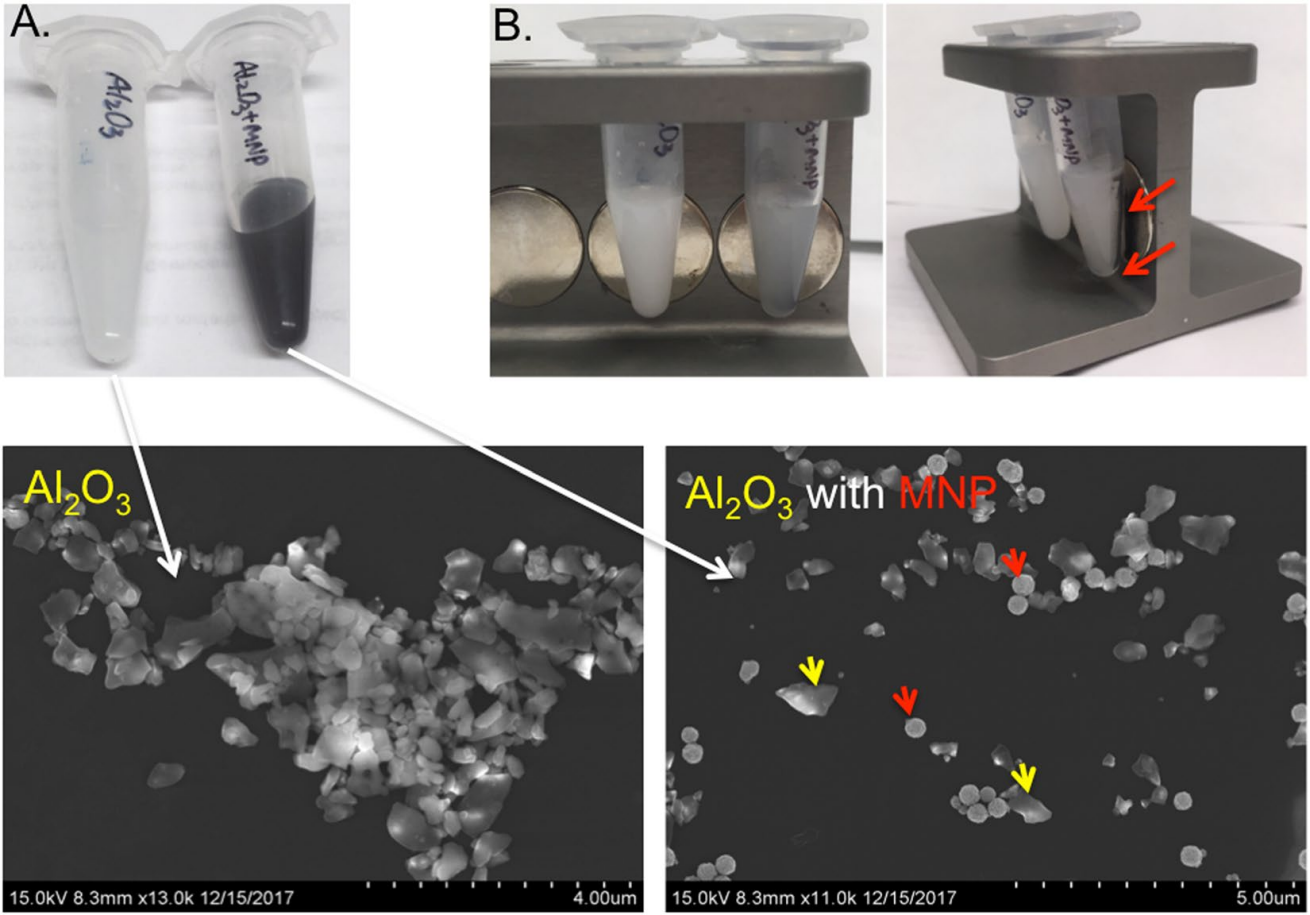
Al_2_O_3_ recognition with Magnetic Nanoclusters coated with MoS2-P15. (**A**) The tubes containing Al_2_O_3_ and Al_2_O_3_ with MNCs solution (**B**) with an applied magnet behind. SEM images represent the solution phase for Al_2_O_3_ and Al_2_O_3_ bound MNPs.

MoS_2_ recognition and recovery by using MNCs were tested with 0.2 mg/500 μl MoS_2_ powder (size in 6 μm) in Milli-Q water (pH 6.9). Two out of three tubes containing MoS_2_ powder were mixed with either 1 mg MNC@APTES@MoS2-P15 or 1 mg MNC@APTES, while the third one used as negative control without any magnetic nanoparticle addition (Fig. 6.A,B). When the external magnetic field was applied, the solution in tube-1 (containing MNC@APTES@MoS2-P15 and MoS_2_ powder) turned transparent, indicating that both the peptide-coupled magnetic NPs and the MoS_2_ powder were completely pulled down by the magnet (Fig. 6C). In tube 2, the MNC@APTES were pulled-down by the magnet but most of the MoS_2_ powder remained suspended in solution, leaving behind a grey solution. Tube-3 containing only an aqueous solution of MoS_2_ powder (while some of this MoS_2_ powder settled under the influence of gravity) remained greyish; indicating that significant amount of the MoS_2_ powder remained suspended. The SEM image of the precipitate in tube-1 clearly showed the MNCs localization on MoS_2_ powder (Fig. 6D). On the contrary, MNC@APTES@MoS2-P15 showed no obvious interaction with Al_2_O_3_ indicating the possible specificity of MNC-peptide system (Fig. 7). These results revealed the ability of MoS2-P15 functionalized MNCs to mine MoS_2_ from aqueous solutions.

Finally, the recovery of MNCs from the MoS_2_ was evaluated. Initially, three tubes containing 0.2 mg/500 μl MoS_2_ powder in H_2_O were mixed with 1 mg nanoparticle of either MNC@APTES@MoS2-P15, MNC@APTES@MoS2-P15(Fmoc) or MNC@APTES@Acetate anhydride. A quick interaction of MNCs and MoS_2_ was observed with MNC@APTES@MoS2-P15 but not with the other nanoparticles. The MNC-MoS_2_ pellet was then incubated with the elution buffer, EB1 (pH 2.2), in order to disrupt the interaction between P15 and MoS_2_ powder and release the MNCs from the powder surface. The visual evaluation of the successful elution was confirmed with ICP-MS analysis of MNC@APTES@MoS2-P15@MoS2 before and after elution. ICP-MS results indicated the complete interaction of MNC@APTES@MoS2-P15 (0.5 mg/mL) with MoS_2_ (0.1 mg/mL) and an almost complete elution of MNC@APTES@MoS2-P15 (1 mg/mL) with trace amount of MoS_2_ (Fig. 6E).

## Conclusion

In this work, we have developed a general scheme for mining MoS_2_ from aqueous solutions using inorganic binding peptides identified from a combinatorial phage display peptide library. Two peptides, namely MoS2-P15 and MoS2-P28 were shown to bind to MoS_2_ trough the interaction between the surface and the specific residues (Val, Pro, Trp), which have hydrophobic and aromatic character. Both of the peptides bind strongly and specifically to MoS_2_ but not to other materials that may be present in the copper mines. The ability of these peptides to capture MoS_2_ under a variety of pH conditions and the stability of their binding is promising for their utilization in harsh environments such as tailing ponds. The smart biomaterial (created by decorating magnetic nanoparticles with the MoS_2_-binding peptides) enabled us to capture and pull down MoS_2_ from aqueous solutions. Furthermore, metal and peptide recovery following their capture was found to be possible, which would allow this biomaterial to be recycled and thus further decrease the processing costs of biomining. The approach we demonstrated here could also be applied to biomining of numerous valuable and rare earth materials. Nevertheless, further studies with real environmental samples and *in situ* evaluations will be required to prove the immense potential of this technology.

## Electronic supplementary material

Supporting Information
