# Supplementary material for: Biomining of MoS2 with Peptide-based Smart Biomaterials
Source: Sci Rep. 2018 Feb 20;8:3374. doi: 10.1038/s41598-018-21692-4 (PMC5820330; doi:10.1038/s41598-018-21692-4)
Supplement: Supplementary file 1 — Supporting Information [file 41598_2018_21692_MOESM1_ESM.pdf]

**Biomining of $MoS_2$ with Peptide-based Smart Biomaterials**

Sibel Cetinel, Wei-Zheng Shen, Maral Aminpour, Prasanna Bhomkar, Feng Wang, Elham Rafie Borujeny, Kumakshi Sharma, Niloofar Nayebi, Carlo Montemagno

**Supporting Information**

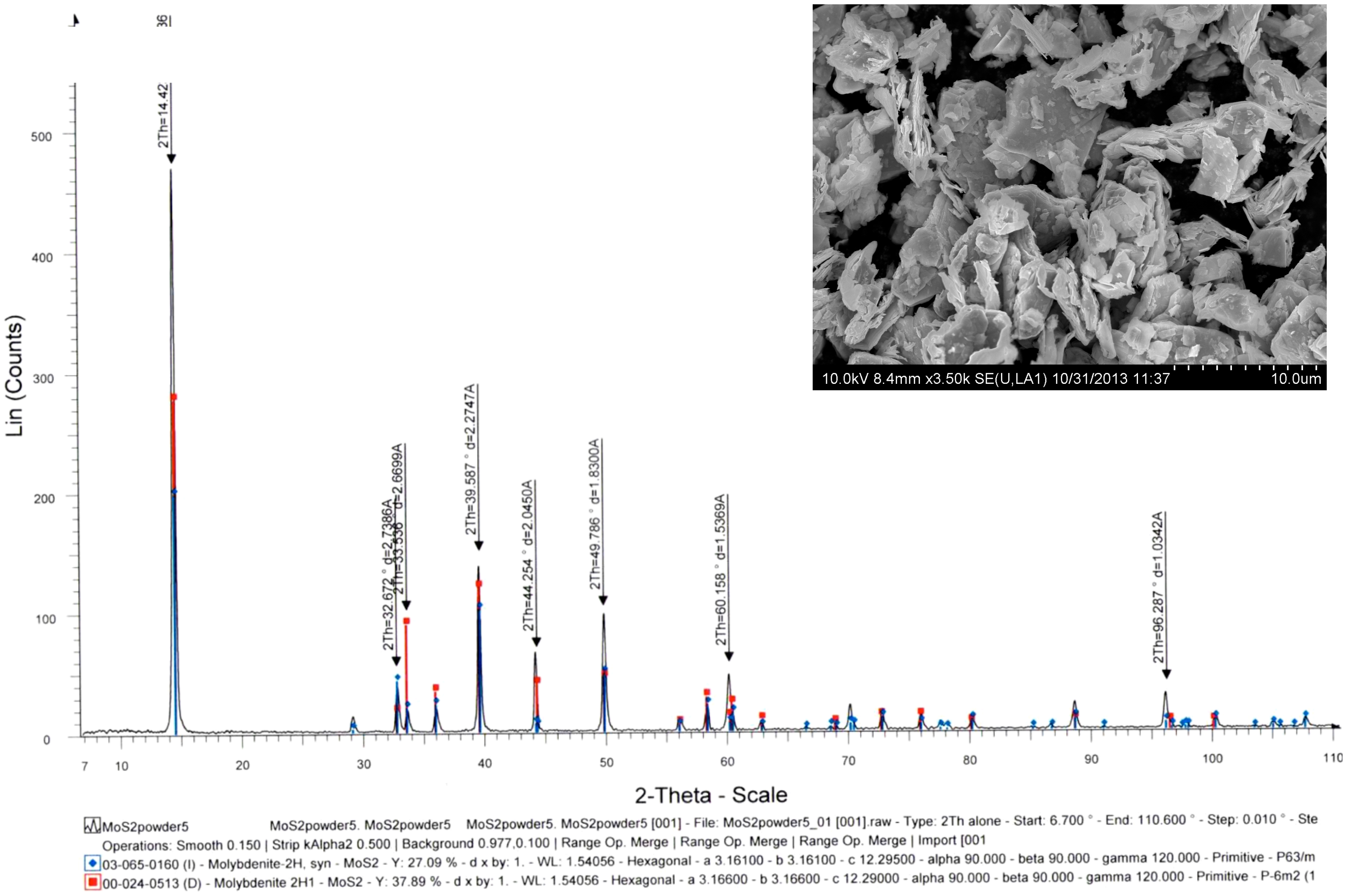


**Figure S1.** $MoS_2$ powder characterization with XRD and SEM

**Method: Characterization of phage binding by fluorescent microscopy**

15 mg of pre-cleaned $MoS_2$ powder was incubated with $10^{11}$pfu phage particles in PBST 0.1% buffer for 3 hours on a rotary cylinder at room temperature. After removal of supernatant (containing unbound phages), the $MoS_2$ powder was washed with 2x1ml PBST (0.1%). Meanwhile, Anti-M13 Monoclonal Antibody (GE Healthcare, catalogue # 27-9420-01) was mixed with Zenan Alexa Fluor 488 Mouse IgG2a secondary antibody (Life Technologies, catalogue # Z25102) at 1:5 ratio and incubated at room temperature for 20 min. 3ul of Ab-dye mix was added to phage-bound-powder and incubated for 10 min at rotary cylinder. Excess Ab-dye mix was washed away with 2x1ml PBST 0.1%. Final phage-bound-powder was resuspended in 500ul PBST 0.1% and analyzed under Olympus IX81 Inverted Fluorescent microscope with under Bright field and FITC filters. The images taken were analyzed with Metamorph Software. Regional statistics of threshold image gave the percent (%) area of the image, which is either the material surface (BF image) or peptide-coated surface (FM image). Phage binding was calculated as a percentage of surface coverage using the equation;

$$\frac{\text{\% Area of FM image x 100}}{\text{\% Area of BF image}} = \text{\% surface coverage (Phage Binding)}$$

Experiments were repeated three times for each phage clone, and a wild-type phage clone was used as negative control.

**Method: Characterization phage binding by spectrophotometry**

15 mg of pre-cleaned $MoS_2$ powder was incubated with $10^{11}$pfu phage particles in PBST 0.1% buffer for 3 hours on a rotary cylinder at room temperature. After removal of supernatant (containing the unbound phages), samples were washed with 2x1ml PBST 0.1%. Both unbound and wash fractions were measured with spectroscopy at 269nm and 320nm. The phage concentrations were calculated by using the equation [1];

$$\text{Phage particles / ml} = \frac{(A_{269} - A_{320}) \times (6 \times 10^{16})}{7222 \text{ (\# of bases / M13 genome)}}$$

Binding affinities were calculated as percent phage bound by using the measured absorption intensities and initial phage concentrations ($10^{11}$ pfu/ml).

Experiments were repeated three times for each phage clone, and a wild-type phage clone was used as negative control.

**Method: Application of Langmuir Adsorption Model to QCM-D measurements**

Based on Langmuir adsorption model, in a QCM-D experiment, following reaction explains the adsorption/desorption process:

$$A + S \underset{k_d}{\overset{k_a}{\rightleftharpoons}} AS$$

where $A$ denotes the free adsorbate, $S$ denotes the free adsorption sites on the surface, and $AS$ denotes the surface bound adsorbate. In addition, $k_a$ denotes adsorption rate constant and $k_d$ denotes desorption rate constant. In a QCM-D instrument, adsorption and desorption are investigated by simultaneous monitoring of the change in resonance frequency ($\Delta f_n$) and dissipation ($\Delta D_n$), with $n$ being the overtone order.

In this work, the raw frequency response (which is denoted by $f$, and is precisely $\frac{\Delta f_n}{n}$) was used to obtain adsorption and desorption rate constants, equilibrium constant, and Gibbs free energy corresponding to the interaction of a peptide and the surface of the gold-coated QCM sensor.

Rate of change of the frequency response was modeled as:

$$\frac{df(t)}{dt} = k_a \left(f_{max} - f(t)\right) C - k_d\, f(t)$$

where $f(t)$ is the response corresponding to occupied sites at time $t$, $C$ is the bulk concentration of adsorbate, and $f_{max}$ is the response for the case that all the binding sites on the sensor are occupied by peptides.

In case that a reference measurement was done using the same background solution prior to each experiment, the initial condition for the frequency was written as:

$$t = 0,\ f(t) = 0$$

Therefore, time evolution of the frequency response was obtained from the solution of the aforementioned differential equation subject to this initial condition as:

$$f(t) = f_{max} \frac{k_a C}{k_a C + k_d} (1 - e^{-(k_a C + k_d)t})$$

If $f_{max} \frac{k_a C}{k_a C + k_d} = f_{eq}$ and $k_a C + k_d = k_{obs}$ was renamed and rewritten as:

$$f(t) = f_{eq} (1 - e^{-k_{obs} t})$$

This equation can be rearranged as:

$$f(t) - f_{eq} = -f_{eq} e^{-k_{obs} t}$$

Therefore, at each concentration, an exponential function was fitted to the experimental $f$ vs $t$ data to obtain $f_{eq}$ and $k_{obs}$. Then, the value of $k_{obs}$ at several concentrations was used to obtain $k_a$ and $k_d$. After that, equilibrium constant was obtained as the ratio of adsorption and desorption rate constants, i.e. $K_{eq} = \frac{k_a}{k_d}$.

**Method: Characterization of Magnetic Nanoclusters Coated with MoS2-P15**

The quantification of peptide functionalization was performed with Bicinchoninic acid (BCA) Assay (Pierce™ BCA Protein Assay Kit) using MoS2-P15 as standard (3500, 1750, 875, 437.5, 217.75, 104.4 and 54.7 μg/ml in PBS). Solutions of MNC@APTES@MoS2-P15 NPs and MNC@APTES NPs were prepared with concentrations 8000, 4000 and 2000 μg/ml in PBS. The amount of functionalized MoS2-P15 peptide on NPs surface was quantified by subtracting the absorbance of MNC@APTES NPs at 562nm from the absorbance of MNC@APTES@MoS2-P15 NPs at three different concentrations; 8000, 4000 and 2000 μg/ml

SEM and TEM images of nanoparticles were taken using a Hitachi S-4800 field emission electron microscope and a JEOL TEM-2200FS transmission electron microscope. Samples for SEM and TEM were prepared by dripping 5μl of dilute sample solution onto a carbon-coated copper grid, blotting excess liquid with filter paper after one minute, and drying at room temperature. X-ray powder diffraction (XRD) spectra were recorded on a XRD-6000 diffractometer in the 2θ range of 15-80$^{o}$ with Cu Kα radiation (λ = 0.154060 nm) and a scanning rate of 0.05 deg $s^{-1}$. X-ray photoelectron spectra (XPS) were acquired on a Kratos AXIS 165 electron spectrometer with 150 W monochromatized A1 Kα radiation (1486.6 eV), where all peaks were referred to the signature C1s peak for adventitious carbon at 284.8 eV. Fourier Transform IR (FTIR) spectra were recorded on a Nicolet 6700 Fourier transform infrared spectrometer in the range of 400-4000 $cm^{-1}$. Field-dependent magnetization was measured on the superconducting quantum interference device (SQUID, Quantum Design, MPMS-XL-7T) magnetometer at 300K.

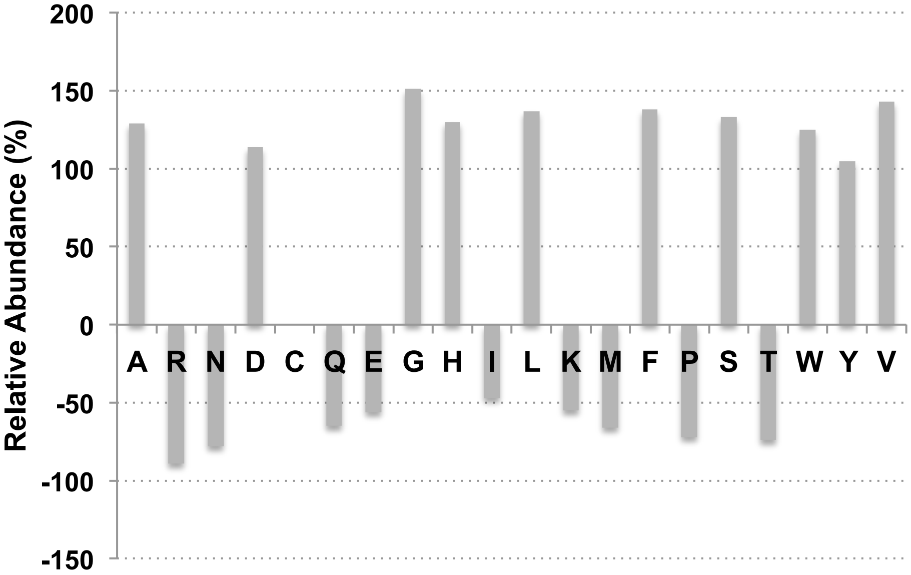


**Figure S2.** Relative abundance of amino acids in selected $MoS_2$ binding peptides

**Table S1.** MW, pI, and Net Charge of Synthesized $MoS_2$ Binding Peptides

| Name | Sequence | Charge | pI | MW |
|---|---|---|---|---|
| MoS2-P15 | GVIHRNDQWTAP | 0.1 | 7.85 | 1393.53 |
| MoS2-P28 | DRWVARDPASIF | 0 | 6.93 | 1432.6 |
| MoS2-P3 | SVMNTSTKDAIE | -1 | 4.07 | 1295.43 |

Amino acids are color-coded based on Lesk Color Code

| | |
|---|---|
| Small nonpolar | G, A, S, T |
| Hydrophobic | C, V, I, L, O, P, F, Y, M, W |
| Polar | N, Q, H |
| Negatively charged | D, E |
| Positively charged | K, R |

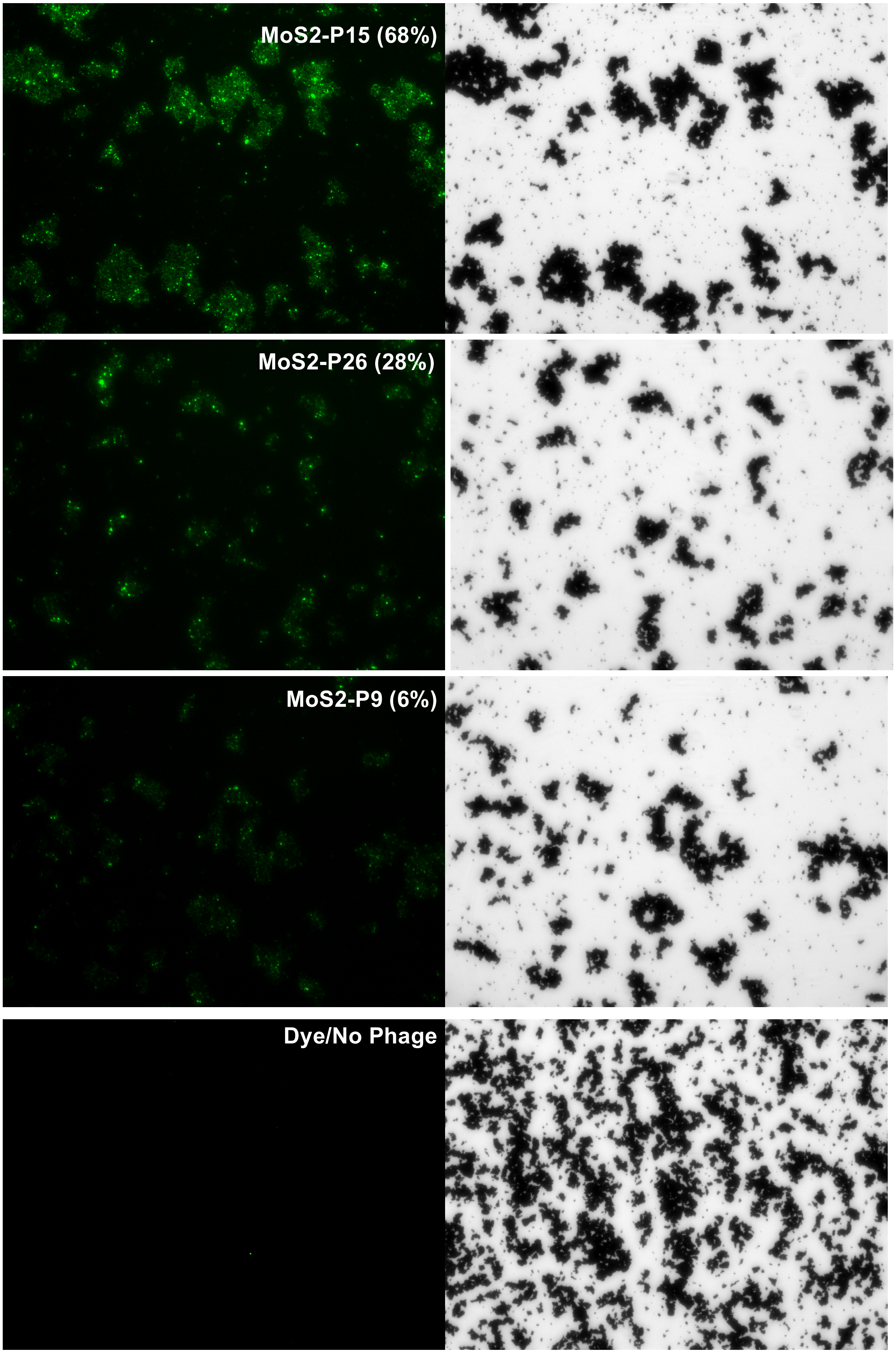
MoS2-P15 (68%)
MoS2-P26 (28%)
MoS2-P9 (6%)
Dye/No Phage

**Figure S3.** Characterization of phage binding by fluorescent microscopy; the pictures on the left represent the fluorescent images while the ones on the right are the bright field. Surface coverage is given for each phage representing the individual peptide namely MoS2-P15, MoS2-P26 and MoS2-P9. No phage control indicates that there is no non-specific dye binding to the $MoS_2$ powder or auto-fluorescence of the powder.

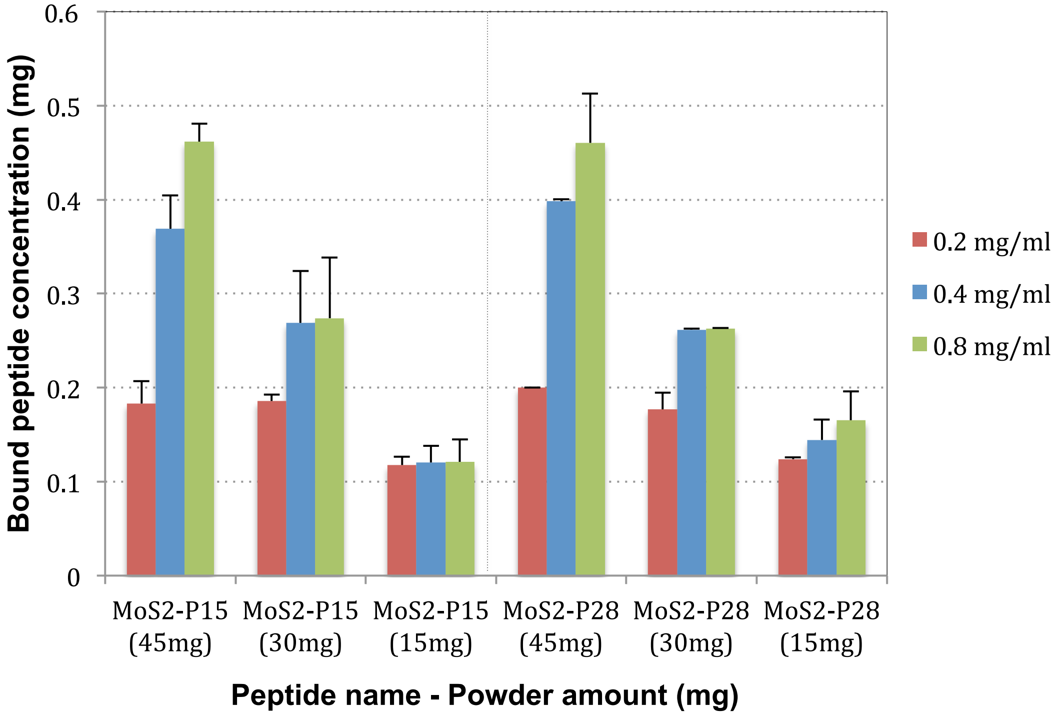


**Figure S4.** Peptide binding

**Table S2.** The specifications of the materials found in tailing ponds and the approximately tested amounts for peptide binding.

| Material | Particle size (um) | Density (g/cm$^2$) | Amount used for binding analysis (mg) |
|---|---|---|---|
| **$Al_2O_3$** | 45-145 | 3.95 | 625 |
| **CaO** | - | 3.3 | 757 |
| **Cu** | ~10 | 8.96 | 27 |
| **$Fe_2O_4$** | - | 5.24 | 500 |
| **Graphite** | <20 | 2.2 | 227 |
| **MgO** | - | 3.58 | 714 |
| **S** | - | | |
| **$SiO_2$** | - | 2.6 | 961 |
| **Tungsten** | ~10 | 19.3 | 12.9 |
| **Zn** | - | 7.133 | 357 |
| **$MoS_2$** | ~6 | 5.06 | 30 |
| **Mo** | ~10 | 10.3 | 30 |

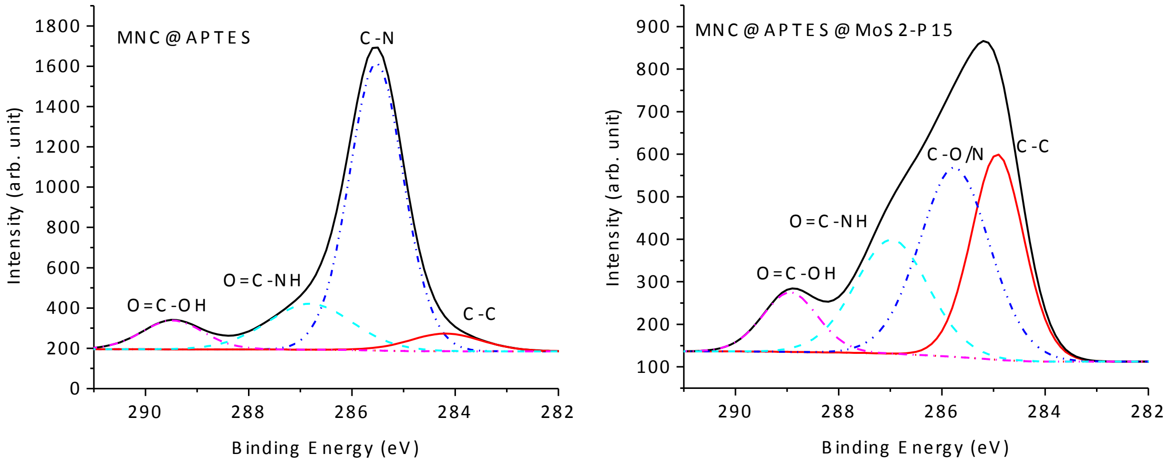


**Figure S5.** XPS spectra of MNC@APTES and MNC@APTES@MoS2-P15

**References**

1. Day LAaW, R.L. (1978) A Comparison of DNA Packaging in the Virions of fd, Xf, and Pf1. In: Denhardt DT, Dressler, D. and Ray, D.S., editor. The Single-Stranded DNA Phages: Cold Spring Harbor Laboratory. pp. 605-625.
